# Environmental Impact of Machinery and Equipment: A Comparison between EXIOBASE, National Environmentally Extended Input–Output Models, and Ecoinvent

**DOI:** 10.1021/acs.est.5c08581

**Published:** 2025-12-09

**Authors:** Yiwen Liu, Meng Jiang, Edgar G. Hertwich

**Affiliations:** † Industrial Ecology Programme, Department of Energy and Process Engineering, 8018 Norwegian University of Science and Technology (NTNU), Trondheim 7491, Norway; ‡ International Institute for Applied Systems Analysis, Laxenburg 2361, Austria

**Keywords:** national accounts, carbon footprints, engines, cranes, logistics systems, electronics, household appliances, manufacturing, robotic

## Abstract

Environmental impact assessments of machinery and equipment (ME) are constrained by process-based life cycle assessment (LCA) with limited system coverage and by aggregated top-down models with reduced representativeness. Lack of knowledge about consistency across these approaches hampers the understanding of ME impacts and policy making. This study quantifies greenhouse gas emission multipliers (cradle-to-gate emissions per unit production) of ME using data from process LCA (ecoinvent), national environmentally extended input–output (EEIO) models, and a multiregional EEIO model (EXIOBASE) for the United States, China, Japan, and South Korea, assessing variations, reliability, and compatibility. While EXIOBASE (seven ME sectors) and national EEIO data (32–102 sectors) broadly align, national EEIO models differ more in production technologies, with deviations from 100-fold lower to 3.7-fold higher than EXIOBASE results. Ecoinvent offers broad ME product-level coverage (∼390 sectors), especially for general and electrical ME, but with uneven representation and limited geographic differentiation. Its multipliers vary widely and often exceed EXIOBASE values, challenging the assumption that process-based LCA underestimates impacts due to truncation. Overall, our results reveal cross-model variation, confirm the relative reliability of EEIO data, point to limitations in ecoinvent, and underscore the need to link technical detail with global trade representation in ME modeling.

## Graphical abstract

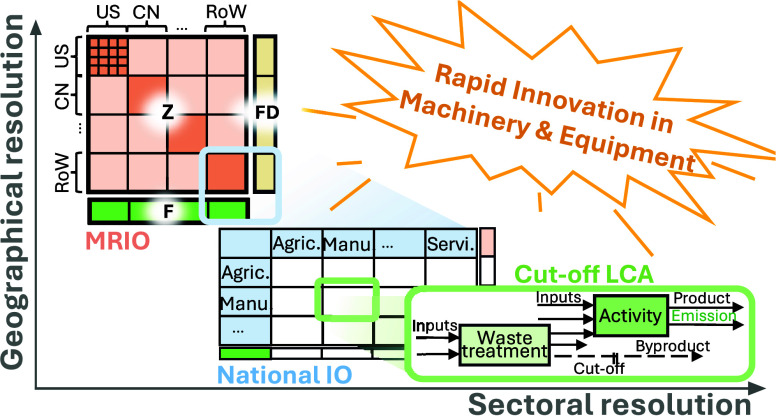

## 1 Introduction

Machinery and equipment (ME), encompassing engines, cranes, logistics systems, electronics, household appliances, vehicles, and other tangible assets that enable productive tasks, operations, and service providing, serve as essential enablers across modern societies. They are the second-largest contributors to greenhouse gas (GHG) emissions^1^ and metal consumption^2^ among manufactured capital goods after buildings, at the global scale. The rapid advancement of automation further amplifies the central role of ME in industrial systems, heightening concerns about their environmental impacts.^2^ These impacts encompass not only GHG emissions and material use but also energy consumption and the growing challenges of e-waste management.

Research on the environmental impacts of ME remains largely confined to the product level. Most contributions rely on process-based life cycle assessment (LCA), focusing on individual products^3,4^ or specific ME categories,^5–10^ predominantly covering electrical machinery,^5,11–19^ electronics and information and communication ME,^20–25^ and general machinery.^6–10,6–10,26–32^ LCA databases such as ecoinvent^33^ offer extensive data, including over 300 ME-related products, enriching analysis at the product scale, though they typically lack accurate production volume data and are not regularly updated. As a result, while valuable for understanding the impacts of individual products or product groups, process-based LCA studies offer limited insights into broader, systematic effects of ME across the economy or its long-term environmental dynamics and help comprehensive scenarios for the future demand for ME and the associated need for resources.

At the macro level, the ME sector is little researched, remaining frustratingly opaque.^34,35^ Most existing research addresses capital goods more broadly,^1,36–42^ often treating ME as a single sector and overlooking their distinct material, energy, and service characteristics. Input–output (IO) tables describe the production of various ME, and some progress has been made in ME-focused studies by combining dynamic material flow analysis (d-MFA) and input–output analysis (IOA).^2^ Jiang et al.^2^ quantified the material and GHG footprints of ME from 2000 to 2019 at the global scale, highlighting the stock changes in different countries. Yet, the results remain constrained by sectoral aggregation, which prevents specifying which types of ME are used by whom and investigating the variation of environmental impacts across different types of ME. This challenge stems from the fundamental trade-off between sectoral detail and regional harmonization within environmentally extended multiregional input–output (EE-MRIO) databases. For instance, EE-MRIO databases such as WIOD,^43^ GLORIA,^44,45^ and EXIOBASE^46^ represent ME with only three, five, and eight sectors, respectively. Prior work has shown that sector aggregation can significantly affect results,^47^ whereas greater sectoral resolution tends to improve accuracy,^48^ particularly in manufacturing.^49^

National IO tables can partly overcome these limitations by offering a better representation of ME production. Some countries regularly publish versions of their national IO tables with varying levels of resolution, including more detailed ME sectors. For example, China’s 2015 national IO table distinguished seven ME-related sectors, while the 2017 and 2020 editions expanded this to 32 and 34 ME-related sectors.^50^ However, the pace of developing high-resolution national environmentally extended input–output (EEIO) models has been uneven: in China^51^ and Japan,^52^ environmental extensions exist only for selected years. Meanwhile, national-level studies of ME’s environmental impacts remain scarce, with a few exceptions such as those assessing GHG emissions from South Korea’s electronics.^53^

Both top-down and bottom-up approaches face systematic limitations for ME. Process-based LCA studies excel in product-specific insights but cannot capture systemic dynamics; EEIO models enable economy-wide assessments but lose detail through aggregation. These challenges are particularly acute for ME, a highly heterogeneous sector encompassing products with widely varying material intensities, lifetimes, technologies, and usage patterns. Current data classifications and reporting remain fragmented and nonstandardized, hindering systematic quantification of their material, energy, and environmental dynamics. In response, research has increasingly sought to integrate approaches^54–56^ for better accuracy and consistency of results and interpretations. This requires understanding and evaluating the quality, availability, and consistency of existing data, prompting emerging comparative studies between process-based LCA data and EE-MRIO models,^49,57–59^ with ecoinvent being the most widely used LCA database.^57–59^ Hybrid EE-MRIO databases compiled in mixed units (e.g., mass units for physical commodities, terajoules for energy flows) are preferred in such comparisons to avoid unit conversion issues,^57–59^ but they typically sacrifice sectoral specificity for broader coverage, limiting their usefulness for detailed ME assessments. Thus, despite methodological advances, ME has remained largely peripheral in comparative studies. Addressing this gap is key as ME constitutes the manufactured capital underpinning industrial systems, and its treatment directly shapes assessments of material and environmental impacts.

Here, we aim to answer two research questions (1): How is the production of ME represented and characterized in EEIO and process-based LCA data sources, and what is a reliable and proper source of data for assessing the environmental impact of ME? By analyzing the differences, we then investigate research question (2): How significant are the variations in GHG emission multipliers within broad ME categories across different sources, and what features influence these differences?

To address these, we systematically analyzed the environmental impacts of ME using three representative sources: EXIOBASE for EE-MRIO-based data, national EEIO model data, which integrates national statistics with high-resolution environmental extensions for major ME manufacturing countries, and ecoinvent for process-based LCA data. We selected GHG emissions as indicators due to their sector-wide relevance, strong data availability, and greater definitional consistency compared to energy use. This type of analysis could also be valuable for assessing energy and material use of ME. We divided our analysis into two comparative layers and compared the magnitudes of GHG multipliers. First, we compare GHG emission multipliers across EEIO data sets to assess differences within top-down approaches. Second, we compare results between EEIO data sets and ecoinvent, evaluating the alignment and divergence between top-down and bottom-up perspectives. Ultimately, this work seeks to better understand the current data and explore the possibility of improving environmental assessments of ME across different quantitative approaches, guiding future methodological development.

## 2 Methodology

### 2.1 Research Framework

#### 2.1.1 ME Scope and Definition

To ensure broad coverage of ME sectors, we selected seven sectors from EXIOBASE to represent ME, covering categories such as machinery and equipment, computers, and transport equipment. This comprehensive definition was designed to capture the full range of ME, following concordance relationships between EXIOBASE and standard classifications^60,61^ and previous research.^2,38,42^ Based on this classification framework, we extracted corresponding products from national EEIO models and the LCA database (ecoinvent) to enable cross-data set comparisons (described in detail in Section 2.2.2). Table 1 presents the complete list of selected sectors, including product numbers from the national EEIO models and ecoinvent.

**Table 1:** ME Product Categories Selected from EXIOBASE, Including the Abbreviation Used in This Study and the Number of Corresponding Products Identified in National EEIO Models and Ecoinvent

			national EEIO products				ecoinvent products			
no.	ME product name	abbreviation	US	CN	KR	JP	US	CN	KR	JP
1	machinery and equipment n.e.c. (29)	general	29	10	23	27	150	151	149	151
2	office machinery and computers (30)	office	5	2	4	6	15	15	15	15
3	electrical machinery and apparatus n.e.c. (31)	electrical	16	7	17	20	156	154	161	160
4	radio, television, and communication equipment and apparatus (32)	communication (Communi. in figures)	5	3	6	7	11	11	6	5
5	medical, precision and optical instruments, watches, and clocks (33)	medical	14	1	6	4	-	-	-	-
6	motor vehicles, trailers, and semitrailers (35)	vehicle	13	3	10	7	52	33	48	48
7	other transport equipment (36)	other transport (OtherTransp. in figures)	12	2	4	8	7	26	11	11

#### 2.1.2 Methodology Overview

We used Global Warming Potential with a time horizon of one hundred years (GWP 100) as the environmental impact indicator. To ensure consistency across sources, we harmonized emissions using the intersection of gases available in all data sets, applying characterization factors from the sixth assessment report of the Intergovernmental Panel on Climate Change (IPCC)^62^ (see Table S1 in Supporting Information SI-1). This harmonization excluded certain gases such as fluorinated gases (e.g., hydrofluorocarbons) due to limited availability, resulting in slightly lower GHG estimates than reported by others.

The comparative assessment of the environmental impacts of ME follows the workflow illustrated in Figure 1. We selected the most recent and high-resolution national EEIO data for major ME manufacturing countries (the United States, China, Japan, and South Korea) along with matching-year data from the EE-MRIO (EXIOBASE) and LCA (ecoinvent) databases. Various data conversions and adjustments were applied to harmonize the structures and enable cross-source comparisons.

**Figure 1:**
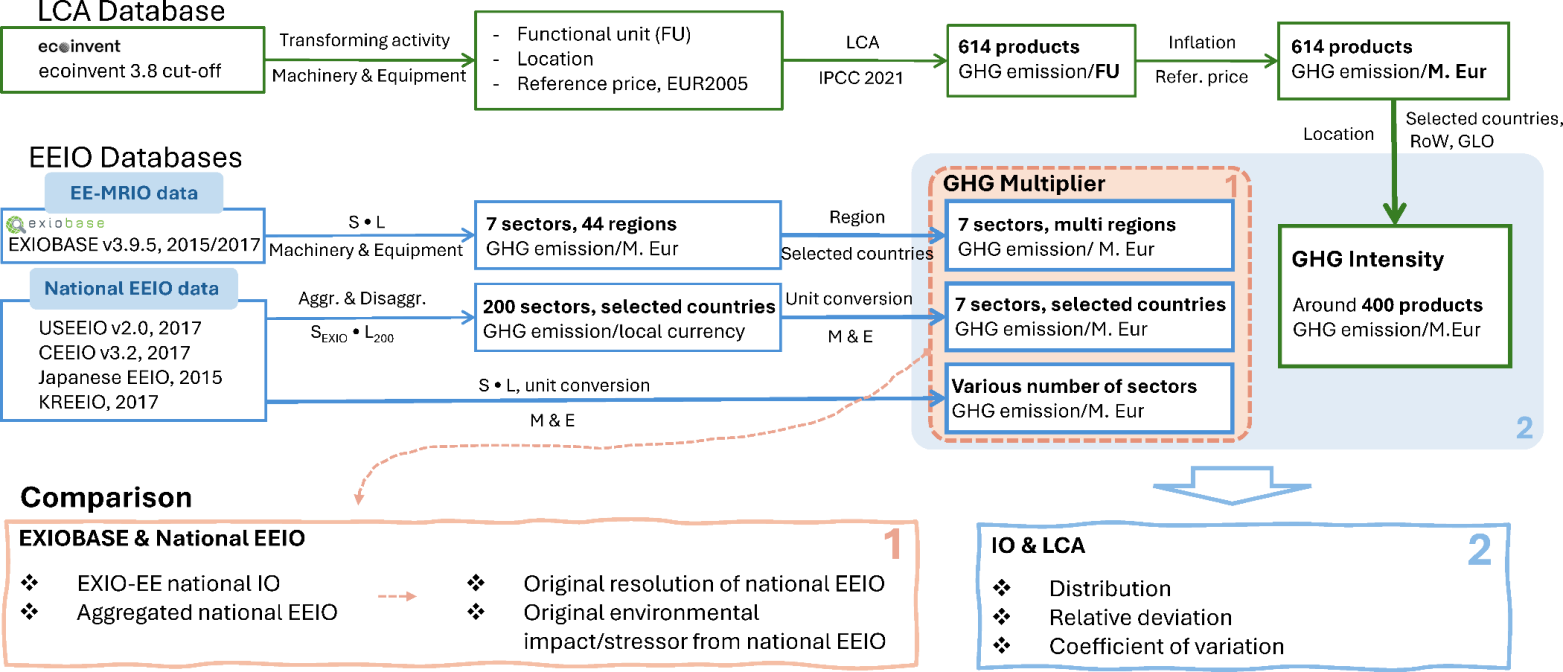
Conceptual framework of methodology. *S* refers to the environmental extension vector (GHG emissions per unit output); *L* is the Leontief inverse matrix. *S*·*L* represents the multiplier calculation. *S*·*L*_200_ represents the multiplier calculation for aggregated national EEIO models and EXIO-EE national IO models at EXIOBASE resolution.

In the first comparison among top-down approaches, we compared environmental impact results between the EXIOBASE and national EEIO models. To assess structural differences and the reasonableness of aggregation levels, we first converted national IO data to the EXIOBASE resolution and applied EXIOBASE environmental extensions, hereafter referred to as EXIO-EE national IO data. We then incorporated the effect of national EEIO environmental extensions by combining converted national IO data with their corresponding converted national extensions, hereafter termed aggregated national EEIO data. Finally, we compared the EXIOBASE data against the original national EEIO data. For EXIOBASE, we distinguished between EXIOBASE global data, which traced impacts along full global supply chains, and EXIOBASE domestic data, which excluded imports and reflect only domestic production. These distinctions are used consistently throughout the paper to avoid confusion and ensure clarity when comparing data sets.

For the comparison between EEIO-based results and process-based LCA results, we retained the original resolution of each data set to examine consistency across approaches and identify opportunities and challenges for improvement. Further methodological details are provided in the following sections.

### 2.2 Data Sources and Harmonization

#### 2.2.1 Data Sources

EXIOBASE v 3.9.5 ^46^ provided the EE-MRIO data and its sector aggregation served as a basis for comparison. It offers time-series data for 49 regions and 200 products, with the version 3.9.5 update calibrated to 2020 and incorporating improved estimates of GHG emissions. National EEIO data were sourced from the latest available statistic IO data for four major ME manufacturing countries^63^ (United States [US], China [CN], South Korea [KR], and Japan [JP]), using the competitive import versions with domestic technology assumption (DTA)^64,65^ and integrated with highest-resolution national environmental extensions from these EEIO models: the US environmentally extended input-output (USEEIO) model (version 2.0)^66^ for the US, Chinese environmentally extended input-output (CEEIO) database (version 3.2)^51^ for CN, the high-resolution environmentally extended input-output model of Korea (KREEIO)^53^ for KR, and embodied energy and emission intensity data for Japan using input-output Tables (3EID)^52,67^ for JP. Although some of the original publications applied these models to specific sectors (e.g., electronics^53^ or healthcare^67^), the underlying models are comprehensive national EEIO frameworks, with their development and validation procedures documented in the respective studies. For JP, where emissions were already reported in CO_2_-equivalents, data were reprocessed based on the National Greenhouse Gas Inventory.^68^ For KR, since the available environmental extension^53^ was already aggregated to GWP100 based on IPCC 1990 report and lacked accompanying emission inventory data, we retained the best available data without reprocessing. Given the approximate share of CH_4_ and N_2_O in the manufacturing sector in 2017,^69^ this may slightly overestimate national EEIO GHG emissions for KR. Ecoinvent^33^ used version 3.8 (cutoff) was selected as the process-based LCA data source.

To ensure consistency in sectoral resolution and temporal coverage, we selected the most recent high-resolution IO data with environmental extensions for each country and selected the corresponding year data from EXIOBASE accordingly: 2017 for the US, CN, and KR, and 2015 for JP. Given that LCA data sets typically represent long-term average conditions, no temporal adjustment was applied to ecoinvent data.

#### 2.2.2 Sector Mapping and Harmonization

We mapped ME-related ecoinvent products to national EEIO sectors, national EEIO sectors to EXIOBASE products, referring the concordance tables for USEEIO and EXIOBASE,^66,70^ the statistical classification of economic activities in the European Community (NACE) rev.2 and EXIOBASE,^60^ and the International Standard Industrial Classification of All Economic Activities (ISIC) and NACE rev.2.^61^ The detailed concordance tables are provided in Supporting Information SI-2. The matching product numbers from national EEIO models and ecoinvent are listed in Table 1. Among the national EEIO models, the US has the highest resolution with 94 sectors, followed by JP (79 sectors), KR (70 sectors), and CN (28 sectors). Electrical and General ME have the highest product counts across all countries. For ecoinvent, Electrical and General ME also have the highest product counts (each ∼150), while Medical ME has no products. In contrast to its high product resolution, ecoinvent offers limited geographic specificity: most entries are labeled as “Global” or “Rest of World”, with only sparse country-specific data for electrical ME (e.g., four for the US, two for JP, and 19 for CN).

For the comparison between EXIOBASE and EXIO-EE national IO models and aggregated national EEIO models, national IO tables and environmental extensions were converted into EXIOBASE format (200 × 200) for *Z* (flow/transactions matrix), *x* (total output), and *F* (GHG emissions) using normalized concordance tables. The normalization process followed this equation, with EXIOBASE data serving as proxies:

Gnew=(G·p+δ)^−1·G·p̂ 1

In equation 1, *G*_new_ represents the normalized, new concordance table and explicitly depends on the *p* vector, which helps disaggregate and distribute the values to more than one destination. *G* is the original concordance matrix between EXIOBASE and national EEIO products containing only 0 and 1; *p* is a weight vector using EXIOBASE data as a proxy, matching the column dimensions of *G* from EXIOBASE data and helping to allocate national EEIO products mapped to more than one EXIOBASE product; and δ is a small perturbation matrix to prevent singularity. The hat represents the diagonalization of the vector. In this study, we calculated four types of normalized concordance matrixes: *G*_make_, *G*_use_, *G*_*x*_, and *G*_F_, using proxies for weight vectors derived from total intermediate consumption, total intermediate input, total output, and GHG emissions of corresponding countries in EXIOBASE (see Figure S2 in Supporting Information SI-1 for schematics). The national IO tables and environmental extensions were converted by eqs 2–4, in which the prime denotes the transpose of a matrix:

$$Z_{\mathrm{NationalEEIO}\text{\_}200}={G_{\mathrm{make}}}^{\prime }Z_{\mathrm{NationalEEIO}}G_{\mathrm{use}}$$ 2

$$x_{\mathrm{NationalEEIO}\text{\_}200}={G_{x}}^{\prime }x_{\mathrm{NationalEEIO}}$$ 3

$$F_{\mathrm{NationalEEIO}\text{\_}200}=F_{\mathrm{NationalEEIO}}G_{F}$$ 4

### 2.3 Multiplier Calculation and Comparative Analysis

#### 2.3.1 GHG Multiplier Calculation

To ensure consistency in comparison and focus on per-unit impacts, we quantified the GHG emission multipliers for ME (cradle-to-gate emissions of unit ME production) from EEIO models and ecoinvent.

For EEIO models, the multipliers were calculated using the demand-driven Leontief model in its environmentally extended form,^71–73^ which links environmental extensions to the production system and traces impacts across the full supply chain:

$$M=s\cdot {\left(I-A\right)}^{-1}$$ 5

In eq 5, *M* represents the GHG emission multipliers; *s* refers to the environmental extension vector, expressing GHG emissions per unit output; *I* is the identity matrix; *A* is the technical coefficient matrix; and (*I*–*A*)^−1^ is *L*, the Leontief inverse matrix.

In this study, we distinguished between domestic multipliers and global multipliers for EXIOBASE. The domestic multipliers were calculated based on domestic data, excluding imports, while the global multipliers accounted for the entire global supply chain, including emission embodied in imports. Meanwhile, the national EEIO multipliers also reflected the environmental impacts along the entire supply chain but with import goods included based on DTA, that is, imports are assumed to have production technologies equivalent to those of domestic products. Additionally, we standardized all monetary values in EEIO models to million Euro (M. Eur) using the annual average exchange rate in corresponding year from European central bank,^74–77^ which also serves as the underlying source for the Eurostat data^78^ used in EXIOBASE.

For ecoinvent data, the GHG emission multipliers were calculated using the open-source Python LCA package, Brightway,^79^ considering only transformation activities of ME in ISIC classification. For unit conversion, we used the reference price data embedded in ecoinvent, combined with inflation derived from UN GDP deflators^80^ to align the unit of LCA results from GHG emission/functional unit to GHG emission/M. Eur. Detailed information about inflation calculations is found in Supporting Information SI-1.

#### 2.3.2 Log_2_ Fold Change

To assess the difference in results, we calculated the quantity change in multipliers for each ME using the log_2_ fold change, as shown in eq 6.

$$\log_{2}\;\mathrm{FC}=\log_{2}\;\frac{M_{\mathrm{target}}}{M_{\mathrm{refer}}}$$ 6

Taking the logarithm ensures a symmetry between the reference and target values, appearing as the same distances on a figure no matter what value serves as a reference. Here, we selected the binary logarithm to reflect multiples of factor 2 differences for its interpretability, and its values can be interpreted directly in terms of “doublings” or “halvings”, which improves clarity.

For comparisons between EXIOBASE and EXIOBASE-resolution national EEIO data, *M*_target_ represents the multipliers derived directly from EXIOBASE data, considering both domestic and global scales, while *M*_refer_ refers to the multipliers calculated from aggregated national EEIO models and EXIO-EE national IO models. This enables the evaluation of differences between results including imports through DTA with results having only domestic production without imports and results including global supply chains. When comparing between EXIOBASE and original national EEIO data, *M*_target_ represents the multipliers calculated from original national EEIO models, and *M*_refer_ corresponds to global multipliers from EXIOBASE. This represents the relative deviation of the differences across national ME and MRIO aggregation proxies.

For comparison between EEIO data and process-based LCA data, *M*_target_ represents the multipliers derived from ecoinvent, while *M*_refer_ is drawn from EEIO databases, i.e., EXIOBASE and national EEIO models, enabling evaluation of resolution effects and consistency across process-based LCA and EEIO systems.

#### 2.3.3 Coefficient of Variation

Unlike EXIOBASE, which provides a single value for each ME, national EEIO models and ecoinvent offer detailed product lists from their respective system perspectives. To assess the representativeness and variation of different products and production technologies across these data sets, we calculated the coefficient of variation (CV), i.e., the ratio of the standard deviation to the mean, for each ME within each data set, following the approach of previous study.^59^

Higher CV values indicate greater variation within a given ME category, suggesting differences in the data granularity and representativeness. Such discrepancies highlight cases where one database may capture more technological diversity than another and point to opportunities for improvement through cross-data set integration.

## 3 Results

### 3.1 EEIO Data Comparison: EXIOBASE vs National EEIO Models

We compare five EEIO-based GHG multipliers: EXIOBASE domestic emissions, EXIOBASE total emissions (including global supply chains), EXIO-EE national IO emissions (using DTA, with emission intensity data from EXIOBASE reflecting supply use structural differences), aggregated national EEIO emissions (using DTA, with converted emission intensity data from national EEIO models to further embed environmental extension impacts), and original-resolution national EEIO emissions (using DTA with original emission intensity data from national EEIO models). Figure 2A shows the multipliers across data sets, with EXIOBASE ranges marked by black lines. Figure 2B shows the differences between EXIOBASE and EXIOBASE-resolution national EEIO multipliers, and Figure 2C illustrates relative deviations between original national EEIO and EXIOBASE global results.

**Figure 2:**
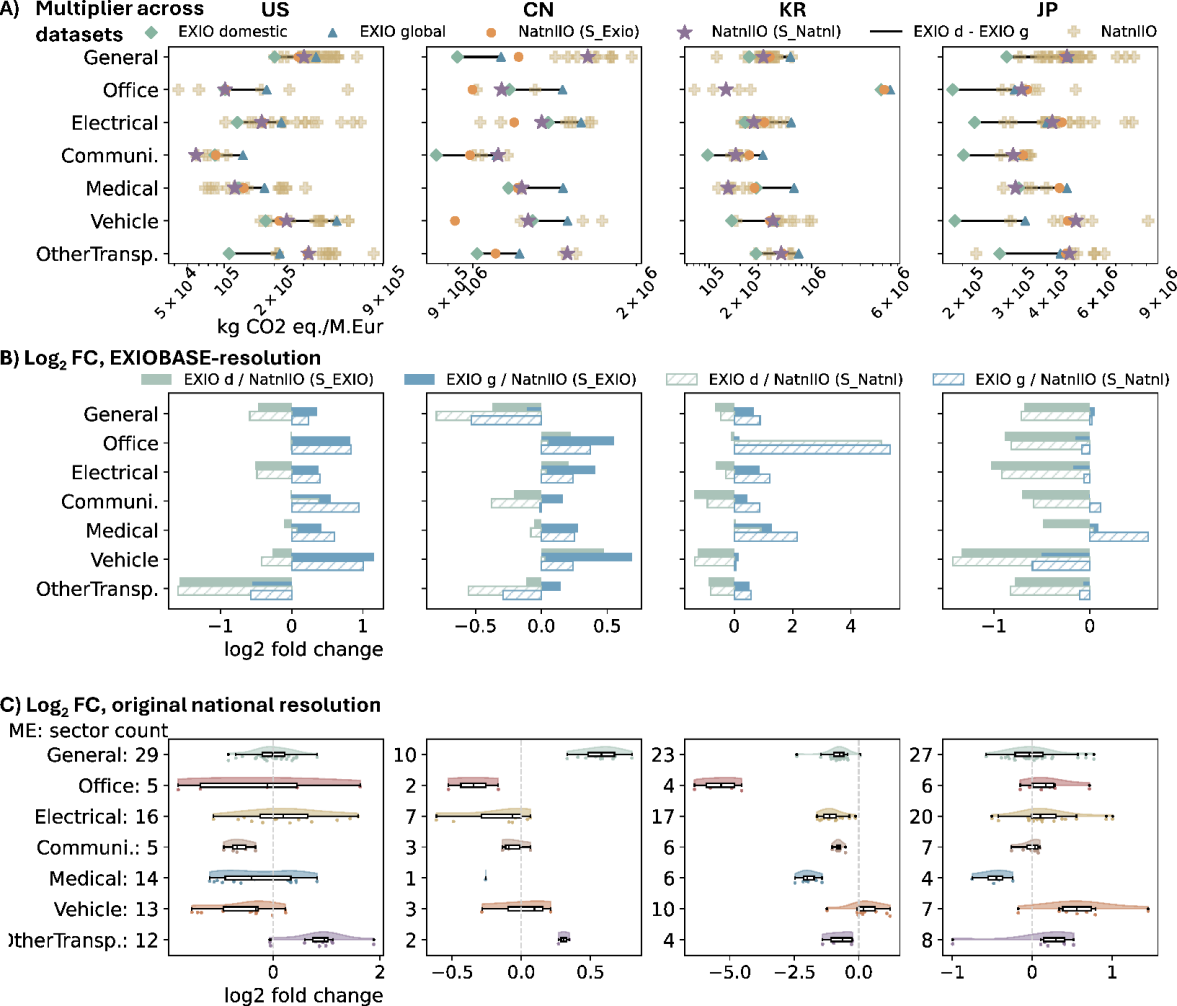
Comparison of EXIOBASE and national EEIO multipliers across selected countries. Rows are for different outcome comparisons and columns for different countries. (A) GHG multipliers from EXIOBASE and national EEIO data with national EEIO multipliers shown in both Exiobase resolution and original resolution. Different markers represent multiplier values, while lines indicate the difference between the EXIOBASE domestic and global results. The orange points represent the EXIO-EE national IO multipliers. The purple asterisks represent the aggregated national EEIO multipliers. The light-yellow cross points represent the original national EEIO multipliers in each ME sector. (B) Log_2_ fold change between EXIOBASE and EXIOBASE-resolution national EEIO multipliers. The legend presents the target value and reference value by *M*_target_/*M*_refer_. (C) Log_2_ fold change between original national EEIO multipliers and EXIOBASE global multipliers (EXIOBASE as a reference). The *y*-axis represents the ME sectors in EXIOBASE, along with the corresponding number of multiplier points derived from original national EEIO data. The raw multiplier data underlying this figure are listed in Supporting Information SI-3.

Across all ME categories (Figure 2A), differences among ME within each country were generally moderate, with no clear cross-country pattern. EXIOBASE multipliers (domestic to global) mostly ranged from 8.7 × 10^4^ to 4.8 × 10^5^ kg CO_2_ eq./M. Eur for the US, 8.6 × 10^5^ to 1.6 × 10^6^ kg CO_2_ eq./M. Eur for CN, 9.6 × 10^4^ to 7.5 × 10^5^ kg CO_2_ eq./M. Eur for KR, and 1.8 × 10^5^ to 4.8 × 10^5^ kg CO_2_ eq./M. Eur for JP. An exception was Office ME in KR, where EXIOBASE intensities yielded significantly higher multipliers (4.8 × 10^6^ and 5.9 × 10^6^ kg CO_2_ eq./M. Eur. for domestic and global, respectively). This discrepancy disappeared in aggregated national EEIO results, indicating a potential error in EXIOBASE extensions. Comparing countries, CN generally had higher ME multipliers than others, with most values exceeding 1 × 10^6^ kg CO_2_ eq./M. Eur for all EXIOBASE, EXIO-EE national IO, and aggregated national EEIO multipliers. In contrast, the US and JP had lower values, and KR reached this threshold only for Office ME. This pattern may reflect both China’s continued reliance on coal for energy, despite progress in reducing GHG emissions,^81^ and the relatively low unit prices for ME compared to those in the other countries.^82^

EXIO-EE national IO results were generally higher than EXIOBASE domestic results (Figure 2A,B), reflecting import inclusion through DTA. However, in CN, multipliers for Office, Electrical, and Vehicle sectors were lower in the EXIO-EE national IO results than in EXIOBASE, suggesting inconsistencies in the supply use structures, which may tend to concentrate in GHG emission-intensive sectors. Considering that national IO tables focus solely on domestic production and consumption structure without the need to balance global trade, we considered them to offer more reliable data. Based on this, further analysis (see Pages S4–S7 and detailed interpretations in Supporting Information SI-1) indicated that, for CN, EXIOBASE may underestimate input requirements for chemicals and nonferrous metals, while overestimating those for basic iron, plastics, and rubber. Similarly, EXIOBASE may underestimate GHG intensities for basic iron, chemicals, glass, and transport services but overestimates those for electricity (coal-based), plastics, and most ME. These mismatches highlighted the need for careful data selection and result interpretation when assessing ME impacts in CN. Likewise, ME studies for KR and JP should be approached with caution, though the multiplier comparison results showed no apparent contradictions, likely due to multiple factors smoothing the results. Corresponding analyses for other countries are provided in Supporting Information SI-1.

Further, when EXIO-EE national IO multipliers exceeded EXIOBASE global values, this implied cleaner production technologies in global supply chains relative to domestic production and vice versa. In the US, CN, and KR, domestic technologies appeared generally cleaner for most ME, except for other transport ME in the US and general ME in CN. However, the findings for CN should be interpreted with caution due to inconsistencies in its supply use structure between EXIOBASE and national IO tables, as previously discussed. In JP, both the EXIO-EE national IO results and aggregated national EEIO results were closely aligned with EXIOBASE global results with log_2_ fold changes well below 0.5, except for Vehicle ME, which showed a cleaner global supply chain. This suggests that domestic production dominates and that national EEIO models are reliable for ME impact assessment in JP. CN also showed strong domestic reliance, with log_2_ fold changes typically below 0.5. In contrast, the US and KR had log_2_ changes above 1.0, indicating greater risks of underestimation when relying solely on the national EEIO data.

The resolution of national IO accounts also influenced the results (Figure 2C). Higher resolutions correlated with broader relative deviations: in comparisons between original national EEIO and EXIOBASE global multipliers, the US (94 sectors) showed deviations ranging from 0.29- to 3.7-fold, JP (79 sectors) from 0.50- to 2.7-fold, KR (70 sectors) from 0.01- to 2.3-fold, and CN (28 sectors) from 0.65- to 1.74-fold. Higher resolutions allowed finer differentiation of ME impacts. Most categories followed approximately normal distributions; however, sectors such as Office ME (US) showed high internal variation having few data points (only five products), indicating strong heterogeneity better captured by national EEIO data but not by EXIOBASE. Outliers, notably in Vehicle (KR, JP) and Other Transport ME (US, JP), reflected aggregation effects that masked obvious technical differences. These results emphasize the value of higher-resolution national EEIO data and suggest that improving ME impact assessments by MRIO could benefit from partial sectoral disaggregation in EXIOBASE, revealing more production characteristics.

Our EEIO-based GHG multiplier comparison reveals differences in data resolution, geographic specificity, and modeling assumptions. Higher-resolution national EEIO data tended to show broader variation and better capture sectoral heterogeneity, whereas EXIOBASE results were more constrained and occasionally misaligned with country-specific realities, as seen in the suspected environmental extension error for Office ME in KR and structural inconsistencies for Office, Electrical, and Vehicle ME in CN. Countries such as JP and CN, which showed strong alignment between EXIOBASE-resolution national EEIO and EXIOBASE global results, may rely more confidently on domestic data for ME analysis when assessing ME impacts at the national level. Meanwhile, the US and KR presented a higher risk of underestimation when relying solely on national EEIO data due to cleaner domestic production. When assessments shift to global supply chain and international trade effects, current MRIO data sets risk masking technical details and introducing potential data bias.

### 3.2 EEIO and Process-Based LCA Data Comparison: EXIOBASE and National EEIO Models vs Ecoinvent

Unlike EEIO data, process-based LCA data are primarily designed for product-level analysis. To compare EEIO and process-based LCA results, we first evaluated EXIOBASE domestic emissions, EXIOBASE global emissions, and ecoinvent results. Due to the absence of reasonable weighting schemes, ecoinvent multipliers were retained at their original resolution. Figure 3A shows the relative magnitude of multipliers; Figure 3B presents log_2_ fold-changes between ecoinvent and EXIOBASE global results.

**Figure 3:**
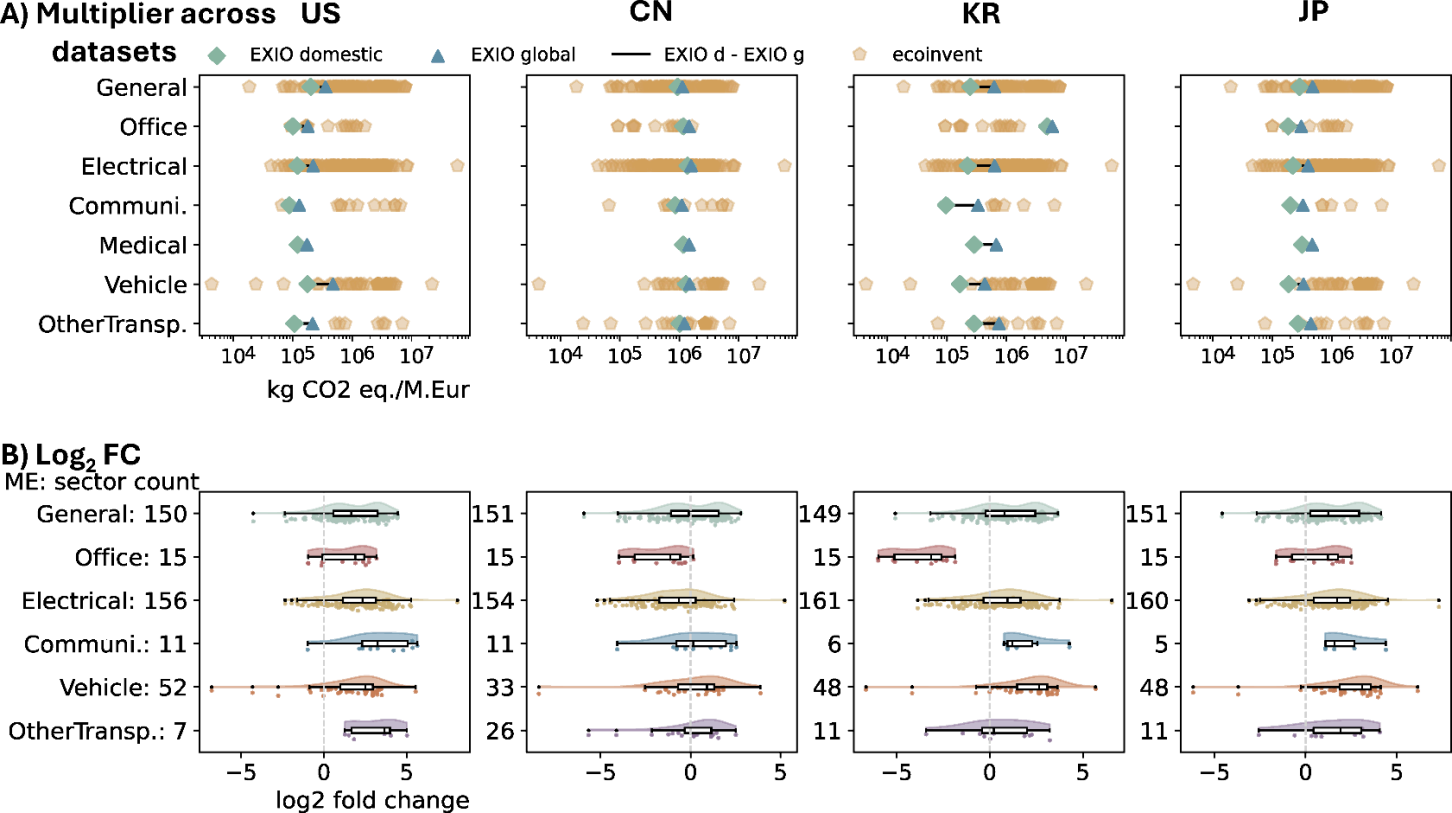
Comparison of EXIOBASE and ecoinvent multipliers across selected countries. Rows for different outcome comparisons and columns for different countries. (A) GHG multipliers from EXIOBASE and ecoinvent data, with ecoinvent multipliers shown in original resolution. The light-yellow pentagon points represent the original ecoinvent multipliers in each ME sector. (B) Log_2_ fold change between ecoinvent multipliers and EXIOBASE global multipliers (EXIOBASE as reference). The *y*-axis represents the ME sectors in EXIOBASE, along with the corresponding number of multiplier points derived from the ecoinvent data. The raw multiplier data underlying this figure are provided in Supporting Information SI-3.

The limited geographic specificity of ecoinvent contributed to the similar distribution of its multipliers across countries (Figure 3A) and limited its capacity to capture regional production differences. Generally, ecoinvent multipliers range widely from below 1 × 10^5^ to over 5 × 10^7^ kg CO_2_ eq./M. Eur, especially in categories like General and Electrical ME, indicating substantial internal variability though no data was available for Medical ME. Most EXIOBASE multipliers fell within this range, except for Office ME for KR, supporting the likelihood of an error in EXIOBASE’s environmental extensions for that category. This pattern of similarity among ecoinvent multipliers was also reflected in the log_2_ fold-change across countries (Figure 3B). While the log_2_ fold-change distributions were broadly similar across countries, deviations varied depending on how EXIOBASE and ecoinvent were compared in each case. Overall, ecoinvent multipliers tended to be higher, possible due to incorrect prices of products in ecoinvent, differences in the ME composition within EXIOBASE ME categories, or the use of data from high-emission countries in ecoinvent. This raises concerns about whether the global ME data in ecoinvent truly reflect average global manufacturing of specific ME, and whether the data set is biased toward high-emission products that may have relatively low production volumes.

Second, we compared national EEIO emissions and ecoinvent results within the EXIOBASE ME categories. Figure 4A plots the relative magnitudes of multipliers; Figure 4B shows distribution histograms, with ecoinvent data distribution in the upper half and the national EEIO data distribution in the lower half; Figure 4C visualizes log_2_ fold-changes between the two; and Figure 4D compares data set representativeness based on total emissions.

**Figure 4:**
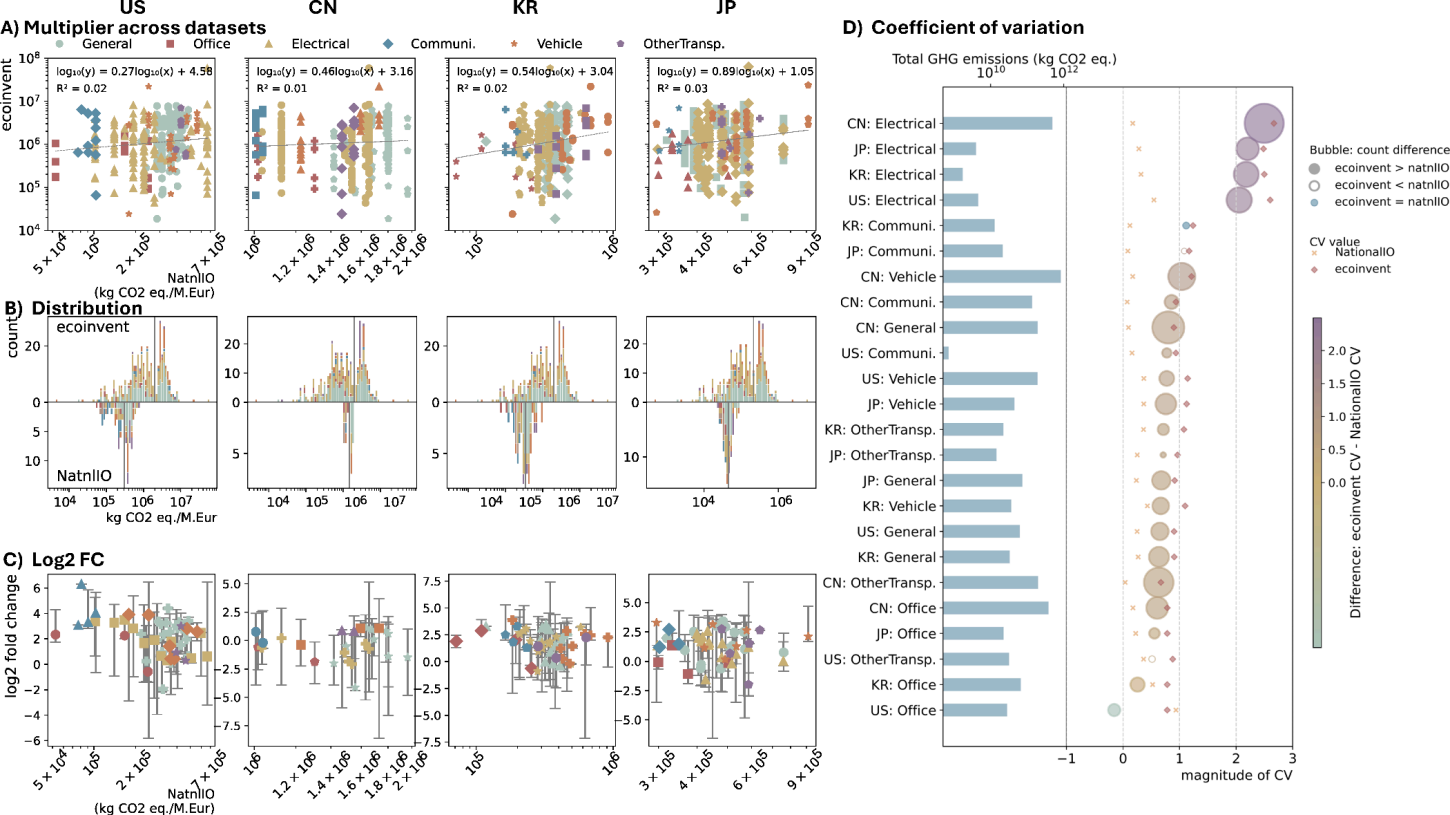
Comparison of the National EEIO and ecoinvent GHG multipliers across selected countries. (A) GHG multipliers from national EEIO and ecoinvent data, with national EEIO multipliers on the *x*-axis and ecoinvent on the *y*-axis, categorized by EXIOBASE ME sectors. The dashed line indicates the fitted line for the log data with the fit expression and *R* ^2^ in the upper left corner. (B) Distribution comparison of GHG multipliers from national EEIO and ecoinvent data, where the *y*-axis represents the count of data points for each data set. The gray line presents the geometric mean result from each data set. (C) Log_2_ fold change between national EEIO and ecoinvent multipliers (national EEIO multipliers as reference) including the distribution of national EEIO multipliers as *x*-axis (median, minimum, and maximum). Subplots in panels (A), (B), and (C) share the same color legend representing EXIOBASE ME sector. (D) Coefficient of variation for results from national EEIO and ecoinvent, sorted by differences. Bubble points on the right indicate CV differences, as shown in the bar legend, with bubble size reflecting the relative differences in data points, calculated as (points_ecoi_ – points_natnl_)/points_natnl_. The absolute CV values for different data sets and ME sectors in each country are also presented for reference. The left bar chart with upper *x*-axis presents the total consumption-based emissions of each ME in 2017 calculating from EXIOBASE. The raw multiplier data underlying this figure are provided in Supporting Information SI-3.

Ecoinvent multipliers showed a wide range even when mapped to national EEIO classifications (Figure 4A). The near-zero correlation between ecoinvent and national EEIO data is surprising. For the data distribution (Figure 4B), ecoinvent showed two peaks (∼7 × 10^5^ and ∼2 × 10^6^ kg CO_2_ eq./M. Eur), with the lower peak mainly driven by General and Electrical ME and the higher peak influenced by General, Electrical, and transport-related ME. Among these, General ME followed the same bimodal pattern, clustering around both multiplier levels, potentially reflecting two distinct internal product groups. In contrast, Electrical ME presented a more evenly distributed pattern, while transport-related ME concentrated at the higher multiplier level. National EEIO data showed clearer country-specific patterns: the US and KR showed broader variation; JP had midrange compression; and CN presented the most concentrated, highest multipliers, possibly because of the small number of categories and hence larger averaging. Consequently, the geometric mean of CN’s national IO data was the closest to that of ecoinvent. These trends suggest that the geographically nonspecific ecoinvent data, likely predominantly sourced from Chinese manufacturers, align better with CN’s national IO data. Nonetheless, using average process-based LCA data may risk overestimating impacts; direct manufacturer-specific data are preferred whenever available. The log_2_ fold-change distributions further confirm the wide range of ecoinvent multipliers in national EEIO classification (Figure 4C). General and Electrical ME remained the focal points of the widest range across all countries. It shows that even for the classification of high-resolution national EEIO models, ecoinvent multipliers vary by several orders of magnitude. Either these categories must be very heterogeneous, or some of the multipliers must be unreasonable.

While ecoinvent offers detailed product-level data, its limited geographic specificity and lack of weighting reduce its suitability for national or global assessments without supporting information. Ecoinvent multipliers frequently exceeded EXIOBASE values, especially in General and Electrical ME, challenging the common understanding that process-based LCA results are lower due to system cut-offs. The outlier in Office ME for KR again confirmed the potential issues in EXIOBASE environmental extension quality. Comparisons with national EEIO data revealed significant internal variations in each sector, even within finer-resolution top-down models. Whether this is due to the real heterogeneity of the sectors or problems with ecoinvent coverage biases or price data remains unclear. Overall, using ecoinvent data needs careful selection and verification, while bridging process-based LCA and EEIO data requires attention to data quality and representativeness, aggregation methods, and consistent weighting to avoid misleading conclusions in ME impact assessments.

Regarding data set representativeness (Figure 4D), ecoinvent generally captured broader technological variability than national EEIO models. Electrical ME presented the largest coefficient of variation (CV) differences (>2) across all countries, highlighting the need for detailed representation in national EEIO models, particularly for CN where Electrical ME also ranked second in total emissions. Communication ME in KR and JP also showed notable CV differences (>1) with the same number or fewer products in ecoinvent, suggesting that higher EEIO resolution alone does not guarantee greater technological differentiation. For most other ME, CV differences were between 0 and 1, indicating modest advantages for ecoinvent. However, exceptions existed. For example, Office ME in the US showed a negative CV difference, implying that the national EEIO model better captured production variability despite fewer products. Total emissions data further emphasized CN’s dominance, with all its ME among the eight highest-emitting categories, alongside Vehicles from the US. This highlights the importance of improving CN’s EEIO resolution^65^ for a more comprehensive understanding of ME. Yet, beyond Electrical ME, the much larger number of product points in ecoinvent did not consistently lead to significantly improved representativeness compared to CN’s national EEIO data (CV difference <1). This underscores the value of including representative products to broaden ME coverage in national EEIO models, as simply increasing the number of sectors is insufficient. Since different production recipes could result in similar emissions, incorporating similar analysis for material and energy use would provide more robust criteria when identifying representative products.

## 4 Discussion

### 4.1 ME in Environmental Impact Modeling

Manufactured capital fundamentally shapes environmental impact assessments because both its production and in-use stocks strongly affect energy, material, and emission flows across the global economy.^83,84^ Prior studies have shown that whether capital goods are treated as final demand or endogenized into production substantially changes footprints, sometimes shifting national responsibilities by tens of percent.^37,38,41,85^ When capital is endogenized in EE-MRIO models (i.e., adding the purchases of capital into the intermediate input matrix), consumption-based carbon footprints increase by 7–48%, sometimes reshaping country- and sector-level responsibilities and modifying observed trade patterns.^37,41^ Our results add to the understanding of ME impact analyses by showing that differences in representation, through sector aggregation, trade assumptions, and environmental extensions, drive variation in the carbon footprint of ME within groups defined by EXIOBASE. This finding frames the more detailed insights that follow: national EEIO models reveal the value of sectoral detail (Section 4.2.1), ecoinvent highlights both the potential and pitfalls of product-level data (Section 4.2.2), and the outlook presents potential improvement of ME impact analyses (Section 4.3).

### 4.2 Potentials and Challenges behind Current ME Data

#### 4.2.1 Insights from EEIO Data

The higher resolution of national EEIO models, capturing sectoral heterogeneity and country-specific structures, clearly adds meaningful information compared to EXIOBASE. Categories that appear as a single ME sector in EXIOBASE often reveal a widespread when broken down in national tables, differing by up to a factor of 3.7. This raises the question: how much is this spread compared to the uncertainty inherent in GHG estimates? For EE-MRIOs, Rodrigues et al.^86^ quantified uncertainties of 2–16% for national consumption-based carbon accounts, with CN as a leading source, while product-level uncertainties reached 50–130% for ME. We can hence see that the differences among types of machinery distinguished in national tables is meaningful even when taking the underlying uncertainty into account.

Even with the spread, the overall picture remains consistent: in most countries, the national EEIO estimates cluster around the corresponding EXIOBASE multipliers. This suggests that EXIOBASE provides a reasonable baseline for the order of magnitude of impacts, while the national EEIO data offer valuable detail to better capture sector-specific differences.

Our findings also suggest that applying DTA can lead to biased estimates, particularly when producing countries rely heavily on imports from regions with very different emission intensities, as observed for the US and KR. This reinforces the need to evaluate emission intensities carefully.

We have identified country-specific challenges in estimating the correct GHG emissions of ME. In CN, inconsistencies can even lead to contradictory outcomes (e.g., EXIOBASE domestic multipliers were higher than EXIO-EE national IO multipliers for Office ME), while in JP, structural differences at the input level largely disappear in multiplier outcomes (Supporting Information SI-1, Pages S4–S7). These cases partially align with earlier findings that uncertainties from environmental extensions can outweigh those from table structures or trade flows.^87^

Although our data do not include explicit uncertainty values, prior work provides useful benchmarks. There is little systematic uncertainty analysis of GHG footprints in the national EEIO models. In our study, the broad alignment between EXIOBASE and national EEIO results in some countries supports robustness, but observed discrepancies highlight the need for transparent documentation, quality control in environmental extensions, and improvements in ME resolution in EE-MRIO databases.

#### 4.2.2 Insights from LCA Database

The LCA database ecoinvent offers the most detailed product-level information on ME, but the variation observed in multipliers is greater than expected. While part of this spread may reflect technological diversity, extreme values suggest artifacts and inconsistencies in the data. For example, the lowest multiplier (3873 kg CO_2_ eq./M. Eur for offshore petroleum platform) likely reflects high estimated infrastructure prices with under-coverage of emission-intensive operations (e.g., material shipping and helicopter transport^88^), whereas the highest value (5.3 × 10^7^ kg CO_2_ eq./M. Eur for scandium oxide for solid oxide fuel cell) is implausible, exceeding the emissions from burning an equivalent economic value of coal. It decreased by ∼78% in ecoinvent 3.11. It remains unclear how much of the variation represents real information versus methodological flaws. This makes interpretation difficult and limits the database’s reliability without careful validation.

The majority of ecoinvent ME multipliers are higher than those from EEIO models. This contrasts with expectations, as IO models typically report higher impacts by avoiding truncation.^89^ Steubing et al.^59^ attributed lower EXIOBASE footprints partly to investment concentrated on several years and neglect of intermediate capital goods. Our focus on multipliers removes the first factor, suggesting deeper inconsistencies. Capital endogenization partially explains the discrepancy: Södersten et al.^37^ found increases of 30–60% for non-OECD and up to 25% for OECD countries, while Font Vivanco^90^ reported up to 60% increases for office machinery, though these adjustments do not fully explain the magnitude observed.

Unit conversions between monetary and physical units further contribute to inconsistency. Roughly 54% of prices in ecoinvent are from input estimates (predominantly Electrical and General ME) that exclude labor, profits, and overheads, which inflate multipliers. Other price sources include UN Comtrade^91^ (∼13%), Simapro (∼8%), and producers or statistics such as Statista^92^ (∼9%). The price source can be checked in ecoinvent data sets using the ME product names listed in Supporting Information SI-3. Previous studies have noted the sensitivity of LCA-IO integration to pricing.^54,93^ Sensitivity analysis showed that relative comparisons remained robust, though absolute levels shifted. Comparison with BACI data confirmed broad reliability, but reinforced the need for stronger uncertainty frameworks. Adjustment methods and corresponding results, and comparative results are provided in Pages S8–S10 in Supporting Information SI-1.

### 4.3 Outlook

Current approaches provide valuable insights into ME carbon footprints, but none of them fully capture the combination of adequate technical detail, global production networks, and country-specific differences that are required for robust and comprehensive assessments. A gap remains in understanding ME carbon footprints, and further investigations are needed to narrow down the uncertainty that we have uncovered.

A feasible way forward is to integrate the technical details of national EEIO data with the trade representation in EXIOBASE, thereby combining production technologies with international consistency. For ecoinvent data, better price information and broader coverage would address some distortions but the wide variation in multipliers suggests that some of the underlying LCA data might be problematic. New LCAs are probably necessary, designed to systematically take into account the entire range of inputs rather than only materials and to capture the prices alongside physical flows.

Such developments would not only reduce uncertainty but also enhance the policy relevance of scenario modeling. This is especially true for circular economy (CE) strategies, which are widely promoted to mitigate environmental impacts.^94^ Also, ME, given their long service lives, plays a crucial role in unlocking CE potential. Bottom-up studies have explored CE opportunities for various ME, including engines,^95^ compressors,^7^ batteries,^96–98^ home appliances,^99–103^ and computers and servers.^14,100,102,104,105^ With improved ME impact assessment, these product-level insights could be linked more directly to large-scale scenario analysis, providing a stronger evidence base for policy and sustainability transitions.

## Supplementary Material







## Notes

SI-1: Characterization factor, extra schematics, and figures; additional results including supplementary figures; expanded discussion on price-based sensitivity analysis (PDF)
SI-2: Concordance tables across EXIOBASE; national EEIO and ecoinvent (XLSX)
SI-3: ME multiplier for each country in each data set (raw data for figures in results) (XLSX)
