# Supplementary material for: Environmental Impact of Machinery and Equipment: A Comparison between EXIOBASE, National Environmentally Extended Input–Output Models, and Ecoinvent
Source: Environ Sci Technol. 2025 Dec 9;59(50):27254–66. doi: 10.1021/acs.est.5c08581 (PMC12750531; doi:10.1021/acs.est.5c08581)
Supplement: Supplementary file 1 [file es5c08581_si_001.pdf]

Supplementary information (SI-1) for

Environmental impact of machinery and equipment: a comparison between EXIOBASE, national EEIO models and ecoinvent

Yiwen Liu*, Meng Jiang, Edgar G. Hertwich

Industrial Ecology Programme, Department of Energy and Process Engineering
Norwegian University of Science and Technology (NTNU)
7491 Trondheim, Norway

*Email: yiwen.liu@ntnu.no

Supplemental Notes. Figures S1-S8. Table S1. 11 Pages.

Table of Contents

Characterization factor....2

Schematics for EXIOBASE proxies....2

Inflation rate calculation....3

Absolute differences between national EEIO and EXIOBASE in L and M, in total and by sectors 4

Sensitivity analysis for price adjustment and price comparison....8

Reference....11

Characterization factor

Table S1 Characterization factor used in this study, based on data from the sixth assessment report of IPCC (IPCC 2022) [1]

**Species**	**GWP100**
CO_2	1.0
CH_4	27.0
CH_4 fugitive and processing	29.8
N_2O	273

Schematics for EXIOBASE proxies

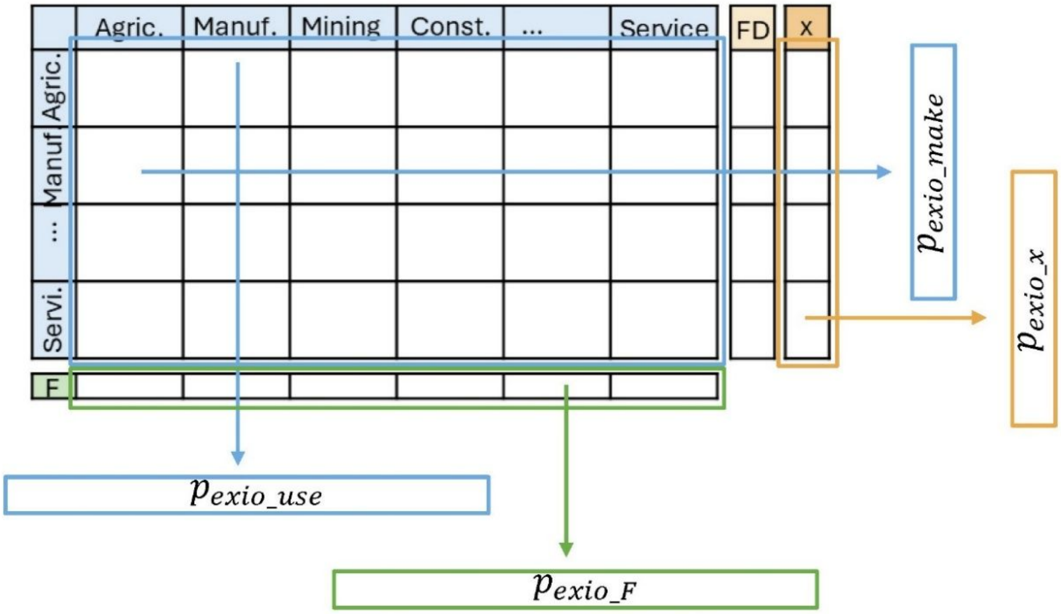

$$
\begin{aligned}
G_{make} &= (G\widehat{p_{exio\_make}} + \delta)^{-1}\, G\, \widehat{p_{exio\_make}} \\
G_{use} &= (G\widehat{p_{exio\_use}} + \delta)^{-1}\, G\, \widehat{p_{exio\_use}} \\
G_{x} &= (G\widehat{p_{exio\_x}} + \delta)^{-1}\, G\, \widehat{p_{exio\_x}} \\
G_{F} &= (G\widehat{p_{exio\_F}} + \delta)^{-1}\, G\, \widehat{p_{exio\_F}}
\end{aligned}
$$

Figure S1 The proxies derived from total intermediate consumption, total intermediate input, total output and GHG emissions in EXIOBASE, with specific equations used for normalizing concordance tables

Inflation rate calculation

Price data embedded in ecoinvent is provided in 2005 Euro. To capture the overall change in price levels across all goods rather than a fixed basket, we applied GDP deflators from the UN Statistical Yearbook (2019)[2] to calculate inflation rates from 2005 to the target years.

$$GDP\ deflator_{year} = \left({GDP_{currentyear}}/{GDP_{constantyear}} \right) \times 100$$

(1)

Equation (1) defines the GDP deflator for a given region and year, where $GDP_{currentyear}$ is GDP at current prices and $GDP_{constantyear}$ is GDP at constant prices. In our study, GDP deflators ere calculated for 2005, 2015 and 2017.

$$Inflation\ rate = \left({GDP\ deflator_{target\ year}}/{GDP\ deflator_{2005}} \right) - 1 \qquad (2)$$

Equation (2) gives the inflation rate relative to the 2005 base year, where the target years are 2015 and 2017.

Due to limited geographic information in ecoinvent (with most products reported at global or "rest of world" scale, and prices expressed in Euro), we applied the European GDP deflator for the majority of products. For products with clear regional attribution (specifically the United States and China in this study), we adjusted prices using country-specific deflators. This was done by (i) converting 2005 Euro prices into 2005 local currency, (ii) applying the local inflation rate based on the corresponding local GDP deflator, and (iii) converting the inflated values back into Euro for the target year.

Absolute differences between national EEIO and EXIOBASE in L and M, in total and by sectors

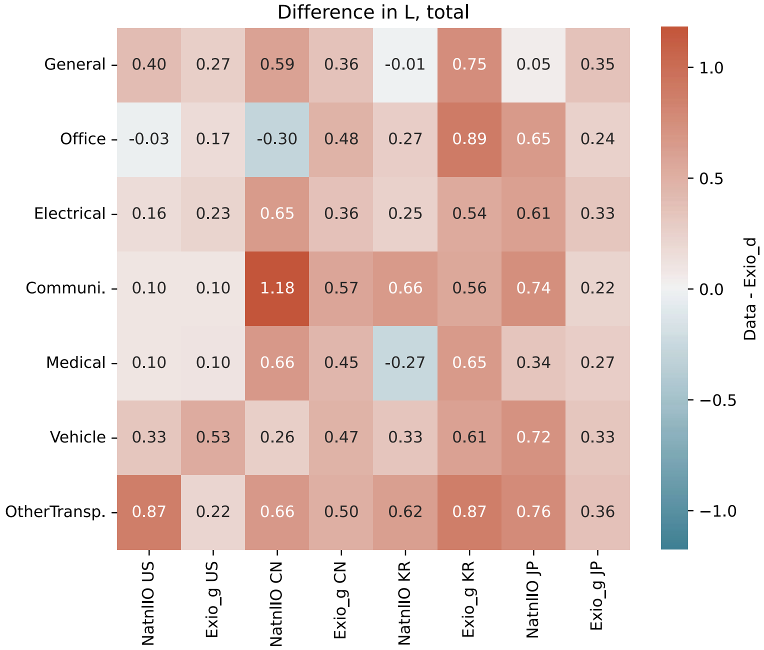

Figure S2 The absolute total differences between EXIOBASE-resolution national EEIO and EXIOBASE in L by ME by heatmap. The number and color present differences between L for EXIOBASE-resolution national EEIO models and Exiobase global data (presenting as x axis) with Exiobase domestic data.

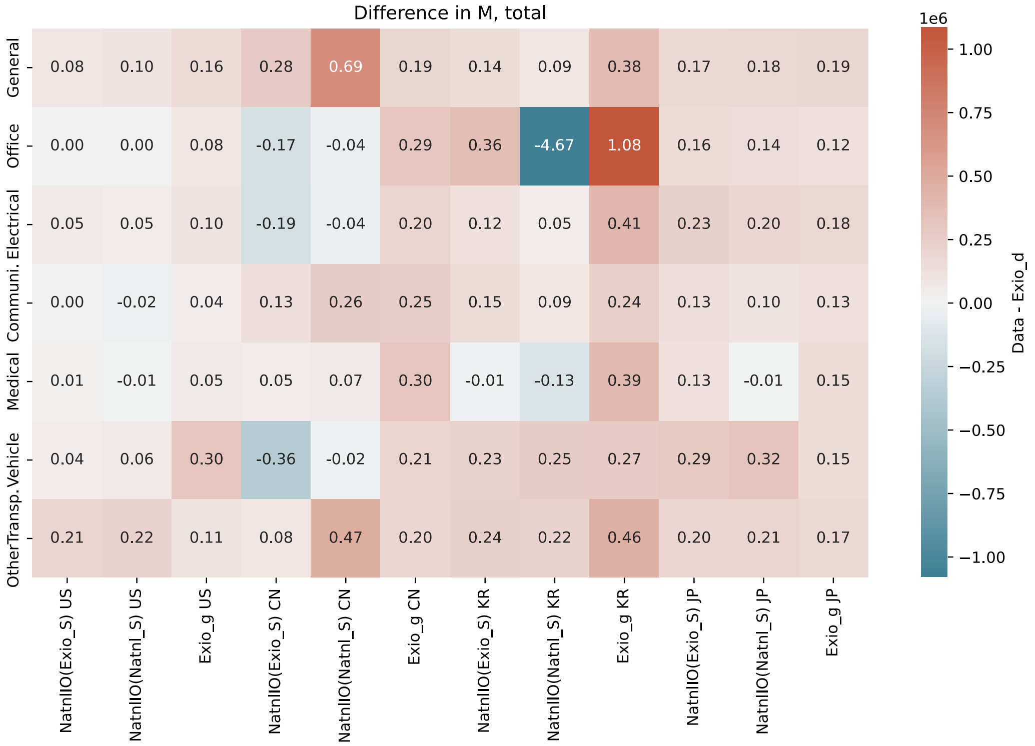

Figure S3 The absolute total differences between EXIOBASE-resolution national EEIO models and EXIOBASE in multipliers by ME by heatmap. The number and color present differences of M between EXIO-EE national IO data and aggregated national EEIO data, and Exiobase global data (presenting as x axis) with Exiobase domestic data.

Comparing total import input requirements (Figure S4), we found distinct country patterns. For CN and KR, imports accounted for a substantial share of ME production in the EXIOBASE models. In CN, the dependency of imports even became higher for most ME except Office and Vehicle ME with DTA. But in KR, the dependency of imports decreased with DTA except Communication ME. The US showed broadly consistent import needs across EXIOBASE and DTA, at least at the total input level. JP displayed a pattern similar to CN, where DTA increased import dependence.

Disaggregating input requirements by sector (Figure S6) highlights clearer mismatches. The US showed the smallest absolute differences between DTA and EXIOBASE, while other countries displayed systematic over- and underestimations. For example, in CN, EXIOBASE appeared to underestimate input requirements for chemicals, non-ferrous metals, and Electrical ME across all ME categories, and for Other Transport ME used in Vehicles and Other Transport ME itself, while overestimating requirements for basic iron, plastics, and rubber. Similar underestimations of Electrical ME and Other Transport ME inputs were observed in KR and JP. This may reflect these countries having similar domestic production technologies but distinct from the global supply chain.

When integrating the impacts of environmental extensions (Figure S5), the total differences in multipliers between EXIOBASE-resolution national EEIO and EXIOBASE were not especially pronounced for CN. Given that ME multipliers in CN were higher than in other countries, the relative gap between DTA and EXIOBASE was less significant, suggesting that national EEIO models provide a reliable basis for Chinese ME impact analysis. However, at sector level (Figure 7), EXIOBASE still appeared to underestimate GHG intensities for basic iron, chemicals, glass, and transport services, while overestimating electricity (coal-based), plastics, and several ME categories. These discrepancies highlight the need for caution when interpreting ME-related results for CN using EXIOBASE. For KR, key discrepancies included overestimation for the intensities of petroleum and natural gas extraction, alongside underestimation for the intensities of basic iron and coal- and gas-based electricity in EXIOBASE. An overestimation was also observed for Office ME intensities, amplifying their multiplier in EXIOBASE. In JP, import differences between DTA and EXIOBASE were largely smoothed out in multipliers, likely because the additional inputs were concentrated in low-GHG intensity sectors such as ME itself. For the US, results were consistently similar across approaches, with relatively small absolute differences compared to domestic results; however, the ability of DTA to fully capture import structures remains better assessed through relative comparisons.

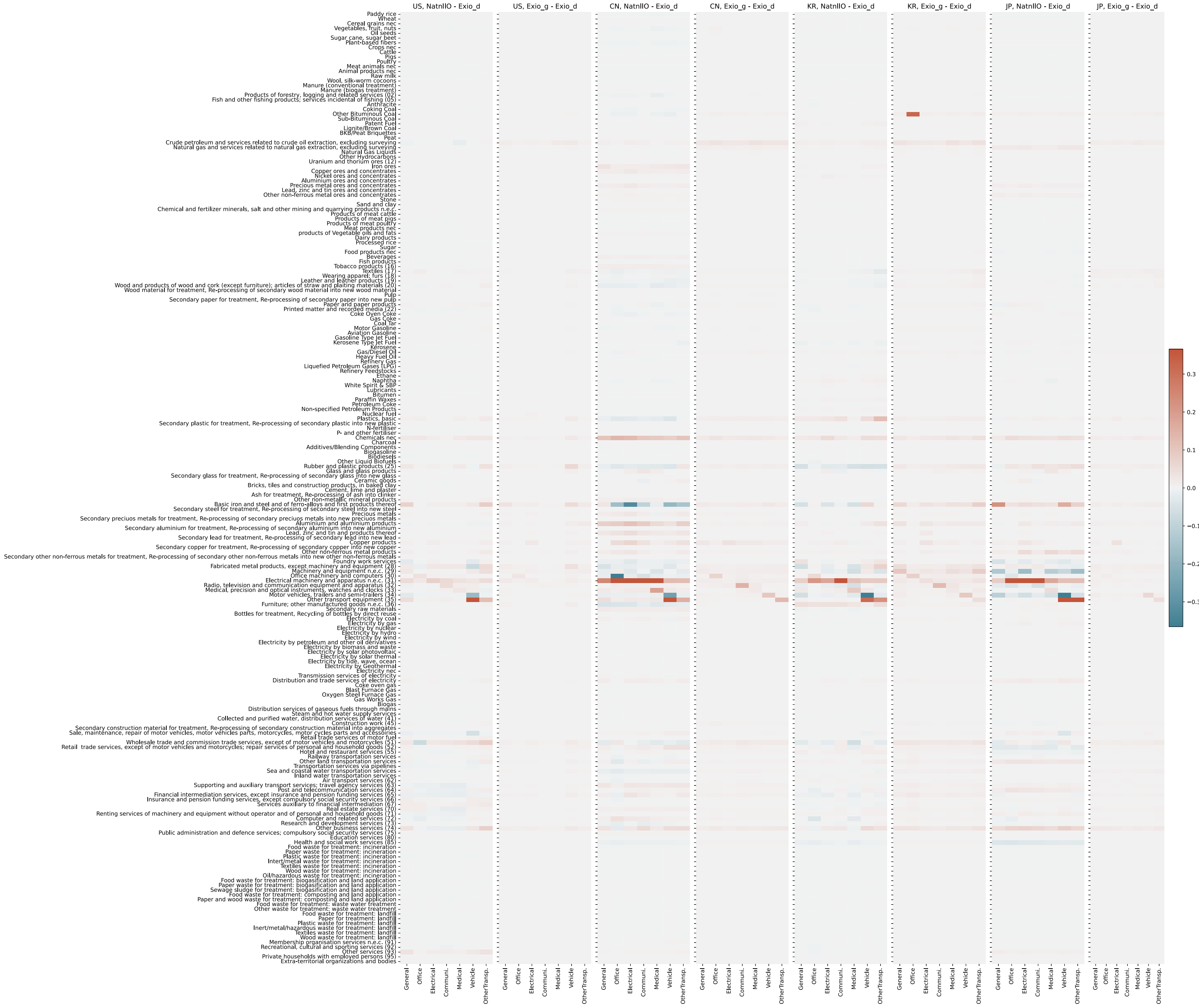

Figure S4 The absolute differences between national EEIO and EXIOBASE in L by MEs and sectors by heatmap. The number and color present differences between L for national EEIO and Exiobase global data (presenting as subtitles) with Exiobase domestic data. The x axis presents ME and y axis presents sectors.

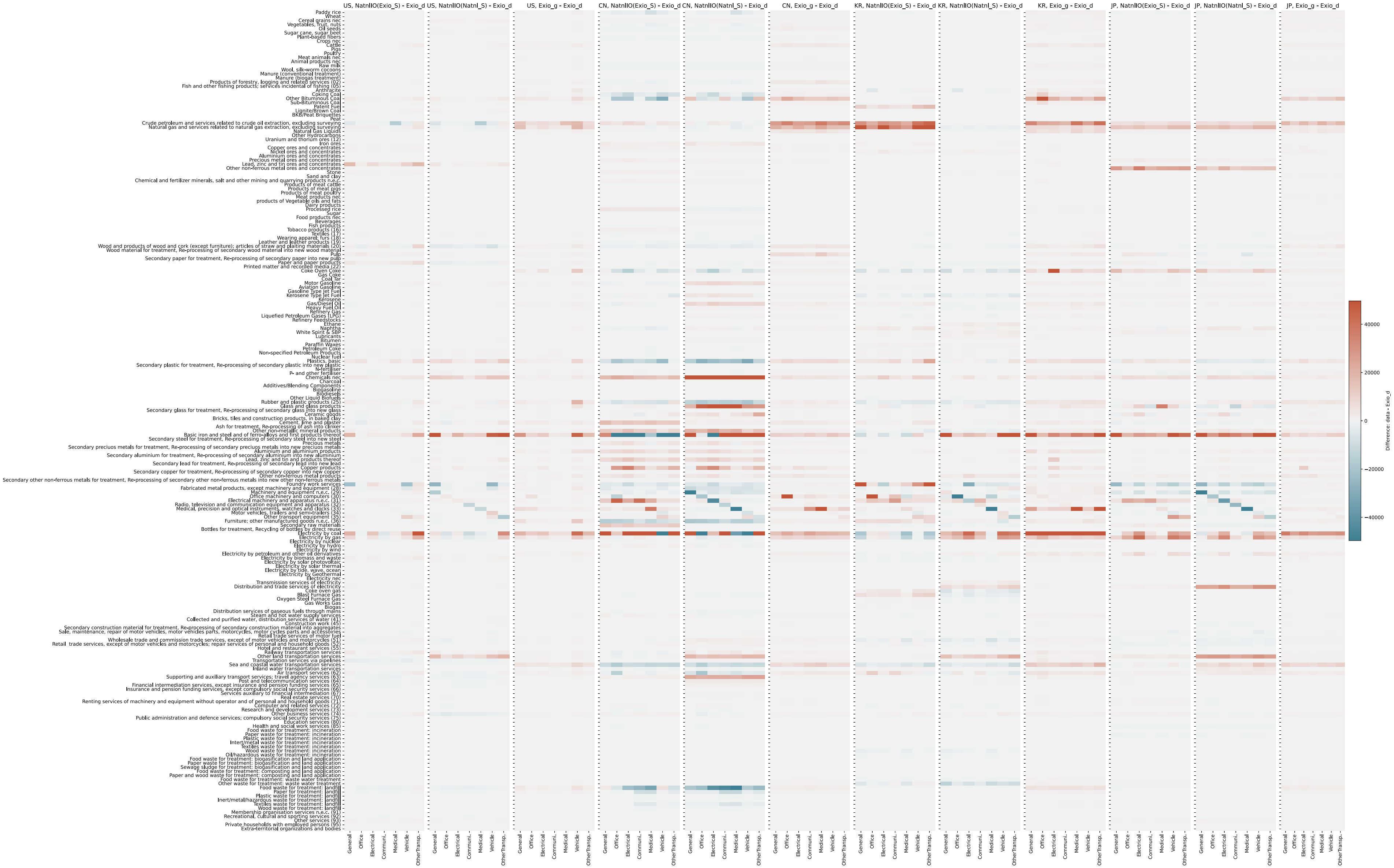

Figure S5 The absolute differences between national EEIO and EXIOBASE in multipliers by MEs and sectors by heatmap. The number and color present differences between M for EXIO-EE national IO data and aggregated national EEIO data, and Exiobase global data (presenting as subtitles) with Exiobase domestic data. The x axis presents ME and y axis presents sectors.

Sensitivity analysis for price adjustment and price comparison

The main concern arises from input-based estimates, which cover around 54% of ME products in ecoinvent (predominantly Electrical and General ME) and risk substantial underestimation due to the omission of additional costs. To evaluate the impact of this underestimation, we performed a sensitivity analysis by adjusting the relevant prices upward by a factor of 2.22 (i.e., 1/0.45), based on the estimated raw material cost shares reported in literature[3]. The results (Figures S8-S9) indicate that while this adjustment narrows internal variation within the ecoinvent dataset and concentrates the distribution (with the exception of Communication ME), the relative comparison to EEIO data remains largely unaffected.

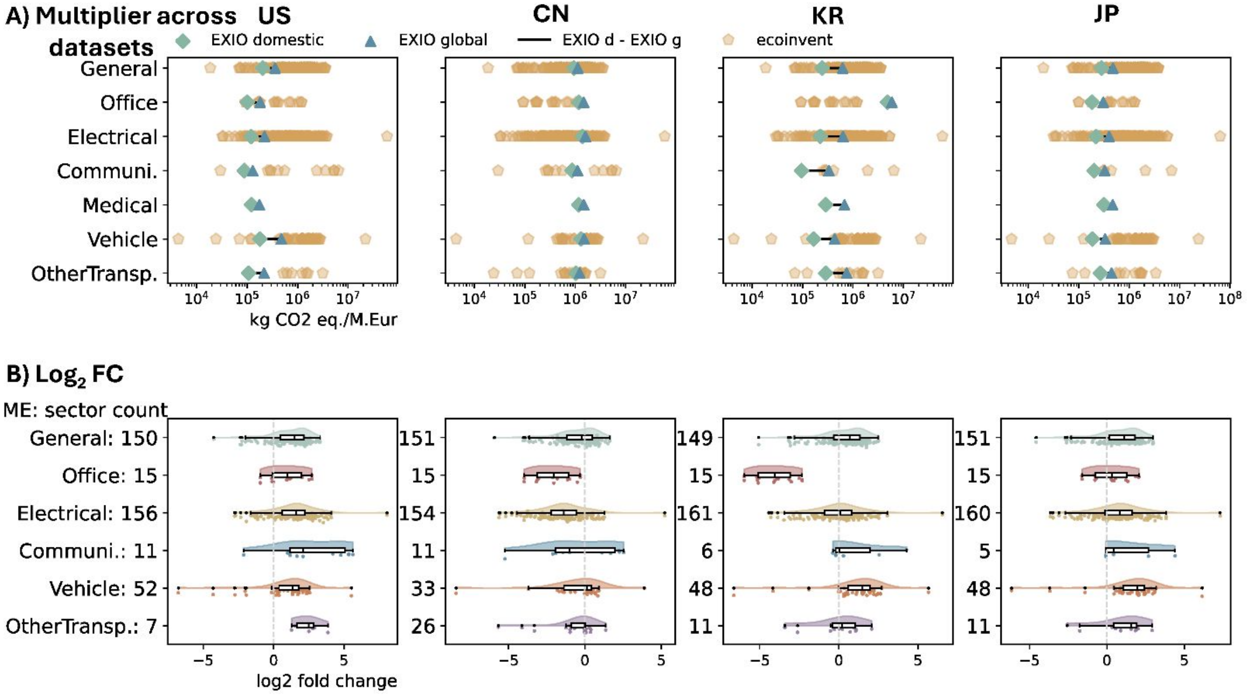

Figure S6 Comparison of EXIOBASE and ecoinvent multipliers across selected countries, with adjusted price estimates. Rows for different outcome comparisons and columns for different countries. A) GHG multipliers from EXIOBASE and ecoinvent data, with ecoinvent multipliers shown in original resolution. The light-yellow pentagon points represent the original ecoinvent multipliers in each ME sector. B) Log_2 fold change between ecoinvent multipliers and EXIOBASE global multipliers (EXIOBASE as reference). The y-axis represents the ME sectors in EXIOBASE, along with the corresponding number of multiplier points derived from ecoinvent data.

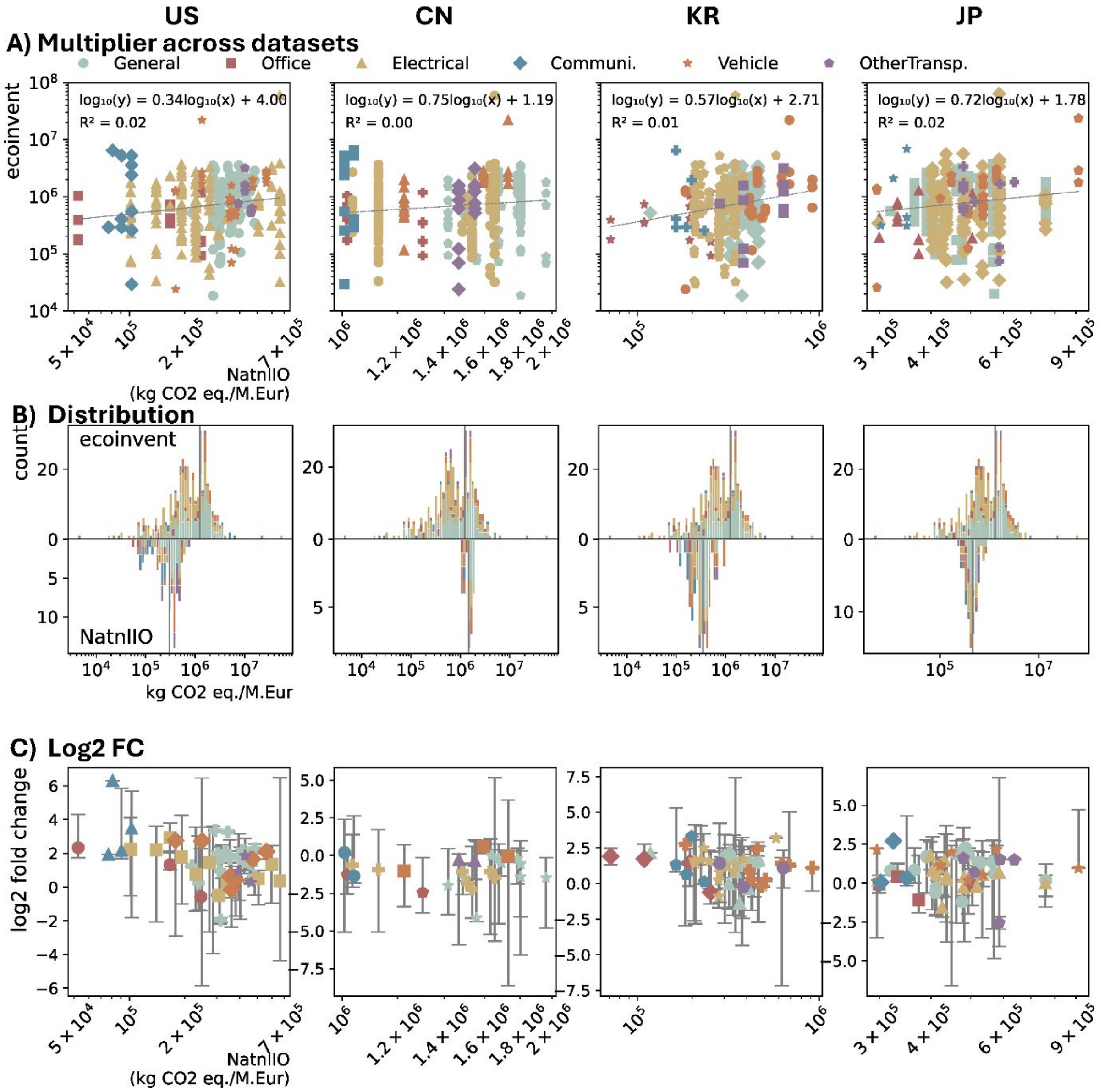

Figure S7 Comparison of national EEIO and ecoinvent GHG multipliers across selected countries, with adjusted price estimates. A) GHG multipliers from national EEIO and ecoinvent data, with national EEIO multipliers on the x-axis and ecoinvent on the y-axis, categorized by EXIOBASE ME sectors. The dashed line indicates the fitted line for the log data, with the fit expression and R^2 in the upper left corner. B) Distribution comparison of GHG multipliers from national EEIO and ecoinvent data, where the y-axis represents the count of data points for each dataset. The grey line presents the mean result from each dataset. C) Log_2 change fold between national EEIO and ecoinvent multipliers (national EEIO multipliers as reference) including the distribution of national EEIO multipliers as x-axis (median, minimum and maximum). Subplots A, B and C share the same color legend representing EXIOBASE ME sector.

Further insights into price uncertainty could be obtained by comparing ecoinvent prices with trade datasets such as BACI, as done in previous studies[4]. Although resolution mismatches between ME in ecoinvent and BACI pose challenges for product-level weighting and aggregation in our case, the available evidence indicates a reasonable degree of consistency between ecoinvent and BACI-derived prices (Figures S10). This suggests that ecoinvent prices are generally reliable within certain bounds but also highlights the need for a more robust uncertainty characterization framework for price data.

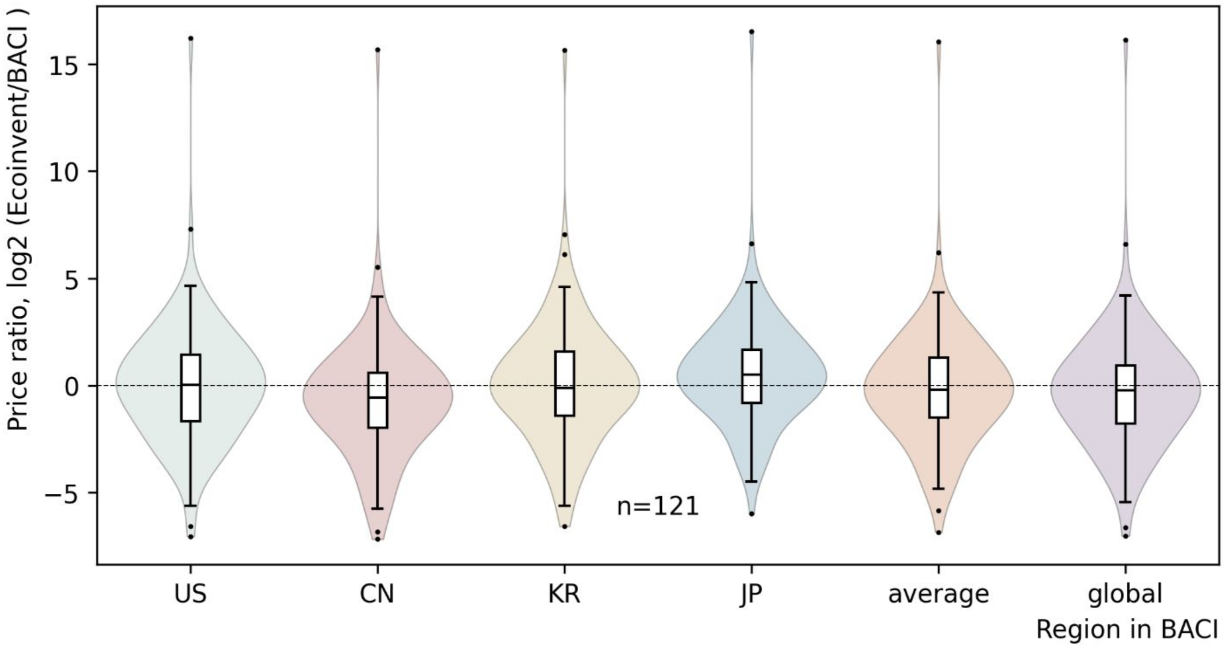

Figure S8 Distribution of price ratios between ecoinvent and weighted BACI data. This analysis includes 121 reference products, a subset of Ecoinvent MEs, limited to those reported in kilograms to ensure unit compatibility. The x-axis indicates the region from which BACI prices were sourced. "Average" reflects the mean across the four selected countries, while "Global" refers to prices based on global BACI data. Each boxplot shows the interquartile range (25th–75th percentile), with the median indicated by the central line.

Reference

(1) Annex II: Definitions, Units and Conventions. In *Climate Change 2022 - Mitigation of Climate Change: Working Group III Contribution to the Sixth Assessment Report of the Intergovernmental Panel on Climate Change*; IPCC, Ed.; Cambridge University Press: Cambridge, 2023; pp 1821–1840. https://doi.org/10.1017/9781009157926.021.
(2) United Nations. *Statistical Yearbook 2019 Edition*; 2019. https://unstats.un.org/UNSDWebsite/Publications/StatisticalYearbook/syb62.pdf.
(3) Wilting, H.; Hanemaaijer, A. Share of Raw Material Costs in Total Production Costs. **2014**.
(4) Jakobs, A.; Schulte, S.; Pauliuk, S. Price Variance in Hybrid-LCA Leads to Significant Uncertainty in Carbon Footprints. *Front. Sustain.* **2021**, *2*. https://doi.org/10.3389/frsus.2021.666209.
